# Causal association and molecular mechanisms of periodontal disease and muscle wasting and atrophy: Mendelian randomization and bioinformatics analysis

**DOI:** 10.3389/fgene.2026.1810047

**Published:** 2026-05-21

**Authors:** Yaxuan Liu, Wenjuan Zhang, Yang Liu, Yuting Bian, Xin Liu, Wei Liu

**Affiliations:** 1 Department of Stomatology, The Second Hospital of Shijiazhuang, Shijiazhuang, Hebei, China; 2 Department of Oral Surgery, The Second Hospital of Hebei Medical University, Shijiazhuang, Hebei, China; 3 Department of Stomatology, The Fourth Hospital of Hebei Medical University, Shijiazhuang, Hebei, China

**Keywords:** bioinformatics analysis, inflammatory cytokines, Mendelian randomization, muscle wasting and atrophy, periodontal disease

## Abstract

**Background:**

Periodontal disease, a chronic inflammatory disorder, is increasingly linked to systemic conditions. This study investigates its causal role in muscle wasting via chronic inflammation, aiming to inform integrative therapeutic strategies.

**Methods:**

Mendelian randomization (MR) analysis was applied to assess causality. Periodontal disease-related genes were obtained from the GEO dataset GSE223924, and muscle atrophy-related targets from GeneCards. Shared targets were analyzed via protein-protein interaction (PPI) networks, followed by functional enrichment (GO/KEGG) and immune infiltration analyses. Key molecular alterations were validated in clinical samples using qRT-PCR and Western blot. Potential therapeutics were identified through drug prediction and molecular docking.

**Results:**

Periodontal disease promotes muscle atrophy through systemic inflammatory dysregulation. We identified 415 shared targets, with IL-6, IL-1β, and IL-10 emerging as core genes. These were enriched in the PI3K-Akt signaling pathway and correlated significantly with altered immune cell infiltration. Experimental validation confirmed dysregulation of these cytokines in patient tissues. Through drug prediction and molecular docking, exploratory potential candidate compounds were obtained. These are only the results of the preliminary virtual screening, including rofecoxib, TT-301 and nelfinavir.

**Conclusion:**

This study initially explored the potential positive causal relationship between periodontal disease and muscle atrophy. This relationship is mediated by chronic inflammation centered on IL-6, IL-1β, and IL-10 within the PI3K-Akt pathway. The findings provide a translational foundation for dual-targeting therapeutic strategies.

## Highlights

• Genetic Evidence for Causal Link: Mendelian randomization provides suggestive evidence for a potential causal association from periodontal disease to muscle wasting and atrophy.

• Core Inflammatory Hub Identified: Identified IL-6, IL-10, and IL-1β as common core targets between periodontal disease and muscle wasting and atrophy.

• Novel Pathway and Therapeutic Implication: The PI3K-Akt signaling pathway is implicated as a potential hub, suggesting novel therapeutic targets for preserving musculoskeletal health.

## Introduction

Periodontitis represents one of the most prevalent chronic inflammatory conditions in dental medicine (Fi and Wo, 2021). Its hallmark pathological feature is the progressive destruction of periodontal supporting structures, clinically manifested as gingival inflammation, periodontal pocket formation, and alveolar bone resorption (Villoria et al., 2024). Epidemiological surveys indicate that the global prevalence of moderate to severe periodontitis in adults ranges from 20% to 50%, with severe cases accounting for approximately 10%, thereby posing a substantial public health challenge (Chen et al., 2021; Jiao et al., 2020). Importantly, periodontitis is not merely a localized oral disorder. Mounting evidence substantiates its strong association with various systemic conditions, including cardiovascular diseases, diabetes, and rheumatoid arthritis (Xiao et al., 2021; Bertolini and Clark, 2023; Hasan et al., 2025). Despite increasing public awareness of oral hygiene, the efficacy of periodontitis prevention and treatment, particularly intervention strategies targeting its systemic complications, requires further optimization.

Skeletal muscle, the primary motor organ in humans, also serves as a crucial metabolic and endocrine tissue (Pedersen, 2019). The preservation of its mass and function is essential for maintaining systemic homeostasis. Muscle atrophy is characterized by a progressive reduction in skeletal muscle mass, volume, and strength, and frequently occurs as a comorbidity in aging and chronic diseases such as heart failure and cancer (Cruz-Jentoft and Sayer, 2019). With the accelerating aging of the global population, the incidence of muscle atrophy and related sarcopenia continues to rise, emerging as a critical public health issue that compromises the wellbeing of the elderly (Petermann‐Rocha et al., 2022). Recent research has established that chronic low-grade inflammation represents one of the central pathological mechanisms that promote muscle protein catabolism and drive the onset and progression of muscle atrophy (Pan et al., 2021; Tuttle et al., 2020). Consequently, investigating the modifiable origins of this inflammatory response has become a key focus in muscle atrophy research.

In recent years, the association between skeletal muscle health and oral health status has gained increasing scholarly interest (Kaymaz et al., 2023). Studies suggest that masticatory impairment in patients with periodontal disease may result in inadequate nutrient intake. Moreover, when periodontal pathogens and their virulence factors enter the systemic circulation, they can elicit a systemic inflammatory response that may directly impair anabolic signaling pathways in skeletal muscle, thereby accelerating muscle atrophy (De Souza et al., 2019; Hajishengallis and Chavakis, 2021). Cross-sectional evidence indicates that clinical periodontal parameters, such as probing depth, exhibit a negative correlation with appendicular skeletal muscle mass index (Song et al., 2023). Nevertheless, observational studies of this nature are prone to confounding by factors such as lifestyle and comorbid conditions, rendering causal inference challenging.

Mendelian randomization (MR), a causal inference approach grounded in genetic principles, employs single-nucleotide polymorphisms (SNPs) associated with periodontal disease as instrumental variables (IVs) to infer causal relationships under specific assumptions (Lu et al., 2025). By examining the association of these genetic variants with muscle atrophy outcomes, MR enables inferences regarding a causal relationship. To ensure validity, the selected genetic instruments must satisfy key assumptions of MR. Recent methodological advances in MR, such as MR-Egger regression and the weighted median estimator, enhance the robustness of causal inference by accounting for pleiotropy, a phenomenon wherein genetic variants influence the outcome through multiple pathways, thereby strengthening the validity of the analytical results (De Ruiter et al., 2025). Elucidating a potential causal association between periodontal disease and muscle atrophy is critical for advancing the understanding of disease mechanisms and may inform the development of targeted prevention and treatment strategies, thereby contributing to improved clinical management.

This study integrates MR imaging with bioinformatics analytical technologies to systematically investigate the causal relationship and underlying molecular mechanisms that link periodontal disease to muscle wasting and atrophy. Initially, MR-based analyses are employed to evaluate the causal association between these two pathological conditions. Subsequent to causality assessment, bioinformatics screening strategies are applied to identify shared molecular targets between periodontal disease and muscle atrophy. Functional characterization of these candidate targets is then conducted via Gene Ontology (GO) and Kyoto Encyclopedia of Genes and Genomes (KEGG) enrichment analyses, aiming to delineate the key signaling pathways implicated in the pathological crosstalk. To validate the expression of key molecular targets, experimental verification is performed using quantitative real-time polymerase chain reaction (qRT-PCR) and Western blotting assays. Collectively, this multi-dimensional approach constructs a comprehensive evidence chain that spans from genetic association to the molecular underpinnings of periodontal disease-related muscle atrophy, providing novel insights into the pathological link between oral and systemic disorders.

## Materials and methods

### Data acquisition and aggregation

A two-sample MR method was employed in this study to examine the causal relationship between periodontal disease and muscle wasting and atrophy. The GWAS Catalog database (ID: ebi-a-GCST90018897) provided the exposure data for this study, which includes summary data from a GWAS on periodontitis involving 348,926 individuals of European descent. Outcome data were obtained from the FinnGen R9 database (ID: finn-b-M13_MUSCLEATROPH), which provides GWAS data on muscle wasting and atrophy phenotypes. The datasets analyzed in this study were de-identified summary data obtained from public sources; therefore did not require ethical approval.

### Selection of instrumental variables

A two-sample Mendelian randomization approach was adopted in this study to assess causality between periodontal disease and muscle wasting and atrophy, rigorously following the three fundamental principles of instrumental variable analysis: (1) Genetic variants were significantly associated with periodontal disease; (2) No influence of these variants on muscle wasting and atrophy through pathways other than periodontal disease; (3) We ensured there was no association between the variants and known confounding factors. Instrumental variables (IVs) were selected using a 7-step standardized procedure in R (v4.3.1) with the TwoSampleMR package: (1). Initial SNP selection: SNPs associated with periodontitis were extracted from the GWAS Catalog (ebi-a-GCST90018897) at a threshold of P < 5 × 10^−6^ to balance statistical power and the number of IVs. (2). LD clumping: SNPs were clumped using the 1000 Genomes Project Phase 3 European reference panel with r^2^ < 0.01 and kb distance >10,000 kb to retain only the most significant SNP per LD block. (3). Palindromic SNP removal: Palindromic SNPs (A/T, C/G) with minor allele frequency 0.42–0.58 were excluded. (4). Horizontal pleiotropy filtering: SNPs associated with muscle atrophy or confounders (*p* < 1 × 10^−5^) in PhenoScanner were removed. (5). Outcome data matching: SNPs were matched to the FinnGen R9 muscle atrophy GWAS (finn-b-M13_MUSCLEATROPH). (6). Instrument strength assessment: Only SNPs with an F-statistic >10 were retained to avoid weak instrument bias. (7). Final IV set: Strictly filtered SNPs were used as valid instrumental variables. To identify instrumental variables, a threshold of (p < 5 × 10–^6^) was adopted, striking a balance between maximizing statistical power and uncovering causal associations. To control for linkage disequilibrium effects, stringent criteria were applied (r^2^ < 0.01 with physical distance >10,000 kb). Instrument strength was verified through F-statistics (all >10), and potentially pleiotropic variants were excluded using the PhenoScanner database (screening threshold *p* < 1 × 10–^5^). The initial SNP screening threshold was set at *p* < 5 × 10^−6^, rather than the stricter genome-wide significant threshold of *p* < 5 × 10^−8^. If the stricter threshold of 5 × 10^−8^ were adopted, it would result in too few instrumental variables and severely insufficient statistical power. This threshold allows for controlling false positives while balancing the number of instrumental variables and statistical power. The F-statistics of all selected SNPs were >10 (mean 22.7, range 20.97–26.7), which met the core criterion of “exclusion of weak instrumental variable bias” recognized in Mendelian randomization studies. Methodologically, it avoided effect value bias caused by weak instrumental variables. Additionally, we performed allele direction harmonization and removed palindromic variants, ultimately obtaining a valid set of instrumental variables meeting all required assumptions. These comprehensive quality control measures substantially enhanced the reliability of our causal inference.

### Mendelian randomization

This study systematically assessed the causal relationship between periodontal disease and muscle wasting and atrophy using several MR methods, including inverse-variance weighted, MR-Egger regression, weighted mode, simple mode, and weighted median approaches. The inverse-variance weighted method was the primary analytical approach, while the other methods were used as supplementary analyses to offer more reliable estimates under various assumptions. This study adheres to the Principled Framework for Mendelian Randomization in Oral Health Research (Bashir et al., 2025).

### Sensitivity analysis

Sensitivity analyses are essential to assess the robustness of MR findings. In this study, the inverse-variance weighted (IVW) method was used as the primary effect estimation, followed by a series of sensitivity analyses. To evaluate horizontal pleiotropy, the MR-Egger intercept test was performed, and MR-Egger regression was used to provide consistent estimates in the presence of directional pleiotropy. Cochran’s Q test was employed to assess heterogeneity across SNP-specific estimates; when significant heterogeneity was detected (*p* < 0.05), a random-effects model was used for the IVW analysis; otherwise, a fixed-effects model was applied (Bowden et al., 2018). To further enhance robustness, we also conducted weighted median estimation (which provides consistent estimates if at least 50% of SNPs are valid) and leave-one-out analysis (to examine whether any single SNP drove the overall result). These complementary methods collectively ensured the reliability of the MR results.

### Identification of periodontitis-associated differentially expressed genes (DEGs)

The original data of the differentially expressed genes (DEGs) related to periodontal disease in this study were sourced from the NCBI GEO database, with the accession number GSE223924. This dataset was sequenced based on the GPL24676 platform and included 20 human gingival tissue samples (10 patients with periodontal disease and 10 healthy controls), which perfectly matched the disease direction of this study. Data preprocessing and normalization: The raw probe-level data were imported into the R software (version 4.3.1), and preprocessed using the DESeq2 package: (1) background correction; (2) quantile normalization (to eliminate systematic errors between samples); (3) converting probes to Gene Symbols and removing unannotated probes; (4) taking the maximum expression value for multiple probes corresponding to the same gene, and obtaining the standardized expression matrix. Quality control and outlier handling: The overall distribution of samples and the separation degree between groups were evaluated through box plots, principal component analysis (PCA), and uniform manifold approximation and projection (UMAP) to verify data consistency. Differential expression gene selection criteria: A linear model for the periodontal disease group vs. the healthy control group was constructed using the DESeq2 package, and the fold change (FC) and significance P value were calculated. The final selection criteria were: after FDR correction using the Benjamini-Hochberg (BH) method, adj.P.Val <0.05, and |log_2_FC| > 0.5. Genes meeting these criteria were defined as periodontal disease-related differentially expressed genes and were included in the subsequent analysis. Periodontitis is a chronic low-grade inflammation. The key inflammatory factors usually show mild but persistent expression changes. When logFC = 0.5, the fold change in gene expression of the treated group relative to the control group is 2^0.5 ≈ 1.41 times. This change range is relatively small and is generally considered a mild regulatory effect, which is consistent with the pathological and physiological characteristics of chronic inflammation in periodontitis.

### Muscle wasting and atrophy target retrieval

Muscle wasting and atrophy-related targets were retrieved from the GeneCards database (https://www.genecards.org/) using the keyword “muscle wasting and atrophy.” Only the targets with a relevance score exceeding 10 were selected. This is the high credibility threshold recommended by the GeneCards platform and is widely adopted in bioinformatics studies of complex diseases to minimize the bias caused by an overly broad gene list while maintaining sensitivity and specificity. These were finalized as muscle wasting and atrophy-related targets.

### Protein-protein interaction network construction

The target lists for periodontal disease and muscle wasting and atrophy were compiled, and a Venn diagram was generated to illustrate their overlap, highlighting potential shared targets between the two conditions. The overlapping targets were subsequently submitted to the STRING database (https://cn.string-db.org/) for the construction of a protein-protein interaction network. In STRING, the “Multiple proteins” option was selected to input the target genes. The species parameter was defined as *Homo sapiens*, and the interaction confidence cutoff was established at a medium level (0.400). To ensure the accuracy of the network, isolated nodes were excluded, and protein interactions were analyzed. The results were saved in JPG and TSV formats.

### Core target analysis

The PPI network was later loaded into Cytoscape to facilitate visualization and topological analysis. Core targets within the network were identified using five algorithms: Edge Percolated Component (EPC), Maximum Neighborhood Component (MNC), Maximum Clique Centrality (MCC), Closeness, and Degree. Core targets within the PPI network were determined by finding the overlap among the top ten genes identified by each algorithm.

### GO and KEGG enrichment analysis

HipLot was utilized to conduct GO and KEGG enrichment analyses, enabling a thorough exploration of the biological functions and signaling pathways linked to the core target genes. A significance threshold was set at an adjusted p-value of less than 0.05. GO analysis categorized core target genes into three distinct functional categories, namely molecular function, biological process, and cellular component. This classification was performed to elucidate the potential roles of these genes in diverse biological processes. KEGG enrichment analysis focused on identifying specific biological pathways, offering deeper insights into the mechanisms driving disease development.

### Immune infiltration analysis

Immune cell infiltration in the GSE223924 training set was profiled using the CIBERSORT algorithm. To ensure reliability, samples with a CIBERSORT output *p*-value >0.05 were excluded. The resulting immune cell fractions were visualized using the ggplot2 package. Differences in immune cell abundance between groups were evaluated with the Wilcoxon rank-sum test. In addition, Spearman’s rank correlation coefficient was computed using the psych package to assess associations between immune cells and key genes.

### Exploratory drug prediction and molecular docking

Candidate compounds with potential therapeutic activity against the selected biomarkers were systematically identified using the Drug–Gene Interaction Database (DGIdb; https://dgidb.org) and the DisGeNET database (https://www.disgenet.org). These computer-based drug prediction and molecular docking analyses are fundamentally exploratory and entirely based on computational methods. A drug–biomarker–disease interaction network was constructed and visualized in Cytoscape to prioritize high-confidence candidates. For molecular docking, the three-dimensional crystal structures of the key target proteins (IL-10, IL-1β, and IL-6) were obtained from the Protein Data Bank (PDB; http://www.rcsb.org). The chemical structures of the candidate compounds, specifically the top five interacting compounds per target as ranked by DGIdb, were retrieved from the PubChem database (https://pubchem.ncbi.nlm.nih.gov). Docking simulations were performed using the CB-Dock2 web server (https://cadd.labshare.cn/cb-dock2/php/index.php) to model the binding interactions between each compound and its corresponding target protein.

### Clinical sample collection

Gingival tissue samples were collected from three patients with periodontal disease at the Stomatological Hospital, along with age- and gender-matched healthy controls (n = 3). Muscle tissues were obtained from three patients diagnosed with muscle wasting and atrophy at the neurology and rehabilitation departments, with three healthy individuals included as controls. All tissue specimens were immediately snap-frozen in liquid nitrogen and stored at −80 °C for subsequent analyses. Peripheral blood samples were also collected from all participants. Written informed consent was obtained from each participant before enrollment, and the study protocol was approved by the Ethics Committee of Fourth Hospital of Hebei Medical University (Approval Number: 2024KS035).

### Quantitative real-time PCR

Total RNA was extracted from periodontal tissue, peripheral blood, and muscle cells utilizing the TriQuick Reagent Kit (SolaiBio, Beijing, R1100). In summary, tissues were either chopped and homogenized in TriQuick Reagent at a ratio of 100 mg tissue per 1 mL reagent or initially frozen using liquid nitrogen prior to the addition of the reagent. Next, chloroform (0.2 mL per 1 mL of lysate) was introduced, mixed thoroughly, and left to incubate at room temperature for 5 min. After centrifuging at 12,000 g for 10 min at 4 °C, the aqueous phase was carefully transferred to a fresh tube. RNA precipitation was achieved by adding isopropanol (0.5 mL per 1 mL lysate), followed by mixing and incubation at ambient temperature for 10 min, and then subjected to centrifugation again at 12,000 g for 10 min at 4 °C. The resulting RNA pellet underwent two washes with 75% ethanol (1 mL per 1 mL lysate) and was allowed to air dry for 5–10 min. Subsequently, the dried RNA was dissolved in 50 μL of DEPC-treated water and incubated at 55–60 °C for 10 min. RNA concentrations were then quantified, and the remaining samples were stored at −80 °C. For cDNA synthesis and PCR, reactions proceeded under these conditions: 25 °C for 5 min, 42 °C for 15 min, 85 °C for 5 min, followed by holding at 4 °C. The amplified products were preserved at −20 °C. PCR amplification consisted of 40 cycles with an initial denaturation at 95 °C for 3 min, followed by denaturation at 95 °C for 15 s and annealing/extension at 60 °C for 30 s. Primer sequences are provided in Table 1.

**TABLE 1:** Primer sequences for target genes.

Gene	Forward (5′-3′)	Reverse (5′-3′)
IL-6	5′-ACT​CAC​CTC​TTC​AGA​ACG​AAT​TG-3′	5′-CCA​TCT​TTG​GAA​GGT​TCA​GGT​TG-3′
IL-10	5′-GAC​TTT​AAG​GGT​TAC​CTG​GGT​TG-3′	5′-TCA​CAT​GCG​CCT​TGA​TGT​CTG-3′
IL-1β	5′-TTC​GAC​ACA​TGG​GAT​AAC​GAG​G-3′	5′-TTT​TTG​CTG​TGA​GTC​CCG​GAG-3′
GAPDH	5′-ACA​ACT​TTG​GTA​TCG​TGG​AAG​G-3′	5′-GCC​ATC​ACG​CCA​CAG​TTT​C-3′

### Western blotting

Western blotting was utilized to evaluate the protein levels of IL-6 (#DF6087), IL-10 (#DF6894), and IL-1β (#AF5103) in tissue specimens obtained from Jiangsu Affinity Biosciences Biological Research Center. The tissues were homogenized in lysis buffer at a weight-to-volume ratio of 1:6 and subsequently centrifuged at 12,000 g for 30 min at 4 °C to obtain the supernatant. Protein concentrations were determined using the Bradford assay. Equivalent protein amounts were separated on a 10% SDS-PAGE gel at 80 V and then transferred onto PVDF membranes using a current of 350 mA for 2 h. Membranes were blocked in 5% non-fat milk for 2 h before overnight incubation at 4 °C with primary antibodies targeting IL-6, IL-10, and IL-1β (dilution 1:1,000), as well as GAPDH (1:5,000). After washing, the membranes were incubated with HRP-conjugated secondary antibodies (1:8,000) for 1.5 h at room temperature. All statistical analyses were performed using GraphPad Prism 6 software.

### Statistical analysis

Statistical evaluations and graphical representations were carried out using GraphPad Prism 6. For data that did not follow a normal distribution, the Mann–Whitney U test was employed, whereas normally distributed datasets were analyzed with a t-test. Additionally, all analyses were replicated in R software using the Two-Sample MR package. Odds ratios (ORs) accompanied by 95% confidence intervals (CIs) were computed, and results with a p-value of 0.05 or less were deemed statistically significant.

## Results

### Instrumental variable selections

After a rigorous selection process, 12 SNPs were identified as eligible instrumental variables. The genetic contribution of each SNP to periodontitis was modest, with individual R^2^ values averaging 6.3 × 10^−5^, and the combined R^2^ for all SNPs was 0.00082, suggests that the chosen genetic variants collectively account for only a small fraction of the phenotypic variation in periodontitis. Instrument strength analysis revealed that the F-statistics for each SNP exceeded the threshold of 10 (mean = 22.7, range: 20.97–26.70), indicating sufficient statistical power of the selected instruments (Table 2).

**TABLE 2:** Instrumental variable selections.

NO.	SNP	F	P val
1	rs117132116	23.1605553843983	1.47299e-06
2	rs117751980	22.78363414	1.88499e-06
3	rs139662047	26.7033326854519	2.35999e-07
4	rs17500377	21.4771809371751	3.60197e-06
5	rs2084850	22.1098777951668	2.58297e-06
6	rs2443514	22.4592285436682	2.15998e-06
7	rs4771666	21.9446375373688	2.80802e-06
8	rs6075314	23.378119733508	1.40799e-06
9	rs6947981	20.973490026947	4.67498e-06
10	rs71640987	23.348293088826	1.357e-06
11	rs9381563	21.7776529502913	3.211e-06
12	rs9995413	22.4478618184533	2.32798e-06

### Examining the potential causal relationship between periodontal disease and muscle wasting and atrophy

**FIGURE 1:**
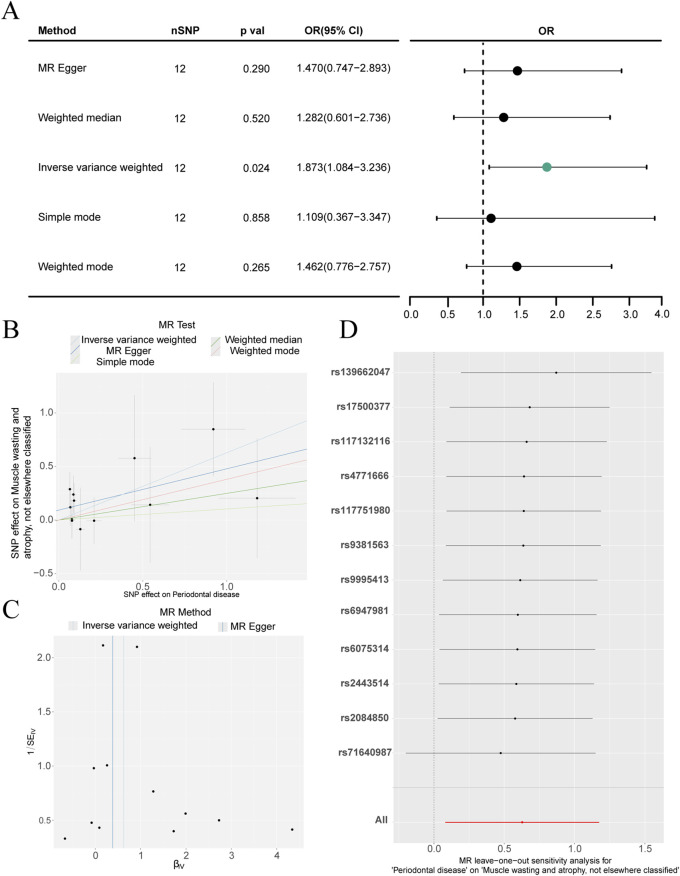
Mendelian randomization (MR) analysis of the relationship between periodontitis and muscle wasting and atrophy. **(A)** Forest plot illustrating the causal effect estimates (Odds Ratios, OR) and 95% confidence intervals (CI) calculated using five distinct MR methods: MR-Egger, Weighted median, Inverse-variance weighted (IVW), Simple mode, and Weighted mode. **(B)** Scatter plot depicting the individual effect sizes of the 12 instrumental single nucleotide polymorphisms (SNPs) on periodontitis (x-axis) versus their effect on muscle wasting and atrophy (y-axis). The slopes of the lines represent the estimated causal effects. **(C)** Funnel plot assessing potential directional pleiotropy; the symmetrical distribution of SNPs suggests an absence of bias. **(D)** Leave-one-out sensitivity analysis demonstrating the stability of the IVW causal estimate after sequentially removing each individual SNP.

As shown in Figure 1A, the inverse variance weighted (IVW) method was used as the main analysis, yielding a statistically significant association between genetically predicted periodontal disease and muscle wasting and atrophy (P = 0.024, OR = 1.873, 95% CI 1.084–3.236). The other MR methods (MR-Egger, weighted median, simple mode, and weighted mode) showed directionally consistent effects but with smaller effect estimates and did not reach statistical significance (all P > 0.05). This approach helped assess whether periodontal disease significantly raises the risk of muscle wasting and atrophy. Specifically, the IVW OR of 1.873 indicates that a one-standard-deviation increase in genetically predicted risk of periodontal disease is associated with an 87.3% higher risk of muscle wasting and atrophy. Results from additional MR methods are as follows: MR-Egger regression OR = 1.470 (95% CI: 0.747–2.893, p = 0.290), weighted median OR = 1.282 (95% CI: 0.601–2.736, p = 0.520), simple mode OR = 1.109 (95% CI: 0.367–3.347, p = 0.858) and weighted mode OR = 1.462 (95% CI: 0.776–2.757, p = 0.265) (Figure 1A). The scatter plot (Figure 1B) demonstrates consistent effect directions across all SNPs, and the funnel plot (Figure 1C) displays a roughly symmetrical distribution of SNP effect sizes, suggesting no strong evidence of horizontal pleiotropy. Sensitivity Analysis.

To verify the strength and reliability of the causal effect estimates from Mendelian randomization, a sensitivity analysis was performed. Heterogeneity testing revealed a Q-value of 6.710 (p = 0.822) for the inverse-variance weighted method and a Q-value of 5.293 (p = 0.870) for the MR-Egger method, indicating no significant heterogeneity among the selected SNPs. The MR-Egger intercept test revealed an intercept of 0.092 (SE = 0.077, p = 0.261), indicating no significant horizontal pleiotropy (Table 3). The small intercept value (0.092), which is close to 0, further strengthens the validity of the causal inference by ruling out significant pleiotropic bias. Furthermore, the MR-PRESSO global test revealed no significant horizontal pleiotropic outliers, thereby reinforcing the robustness of the primary IVW findings. Moreover, the stability of the MR results was systematically tested using a Leave-one-out analysis. As shown in Figure 1D, the significant association between periodontal disease and muscle atrophy persisted following the sequential removal of each individual SNP.

**TABLE 3:** Sensitivity analysis of mendelian randomization.

Exposure	Outcome	Pleiotropy			Heterogeneity					
		Pleiotropy egger			MR-Egger			Inverse-variance weighted		
		egger_intercept	se	P	Q	Q_df	P	Q	Q_df	P
periodontal disease ebi-a-GCST90018897	muscle wasting and atrophy finn-b-M13_MUSCLEATROPH	0.092	0.077	0.261	5.293	10	0.870	6.710	11	0.822

### Identification of shared candidate genes for periodontal disease and muscle wasting and atrophy

**FIGURE 2:**
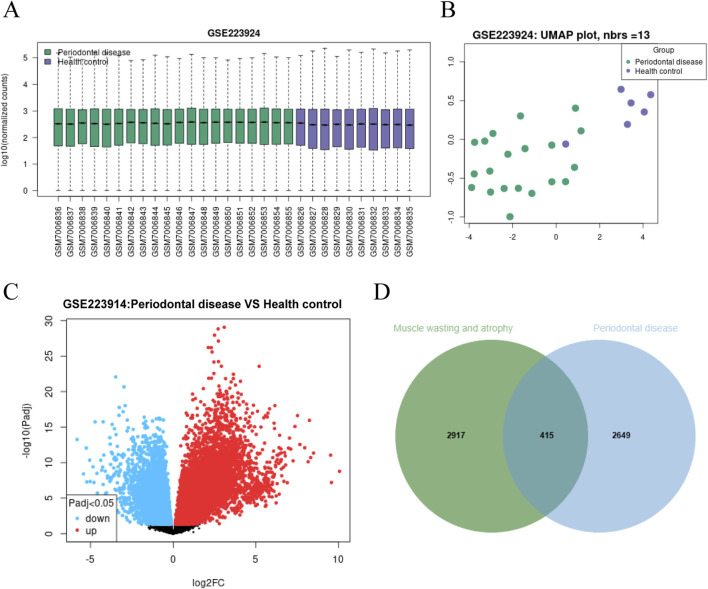
Bioinformatics and differential gene expression analysis. **(A)** Box plot displaying the distribution of normalized gene expression data between the periodontitis group and the healthy control group. **(B)** UMAP plot illustrating the clustering patterns, where green dots represent periodontitis samples and purple dots represent healthy controls. **(C)** Volcano plot comparing gene expression differences between the two groups. Red dots (up-regulated) and blue dots (downregulated) represent significantly differentially expressed genes (DEGs) identified using strict thresholds (Padj <0.05 and |log_2_FC| > 0.5). **(D)** Venn diagram illustrating the intersection of periodontitis-associated DEGs and muscle wasting/atrophy-related targets, identifying 415 shared candidate genes.

We analyzed gene expression differences between the periodontal disease and healthy groups to explore the link between periodontal disease and muscle wasting and atrophy. Initially, we performed a systematic analysis of the periodontal disease dataset (GSE223924). The box plot indicated that the median lines of all samples were aligned on the same level (Figure 2A), suggesting overall consistency in the data. PCA revealed significant separation between the control and periodontal disease groups (Figure 2B). DEGs were displayed in a volcano plot (Figure 2C), providing a clearer visual representation of the gene expression differences. A total of 3,064 genes with a log fold change (LogFC) greater than 0.5 were selected as periodontal disease -related genes (Figure 2C). Following the differential expression analysis, we proceeded to identify potential targets related to muscle wasting and atrophy. Using the GeneCards database, we retrieved 3,332 muscle wasting and atrophy-related genes with a score greater than 10. A Venn diagram identified 415 common targets linked to both periodontal disease and muscle wasting and atrophy (Figure 2D).

### GO and KEGG enrichment analysis

**FIGURE 3:**
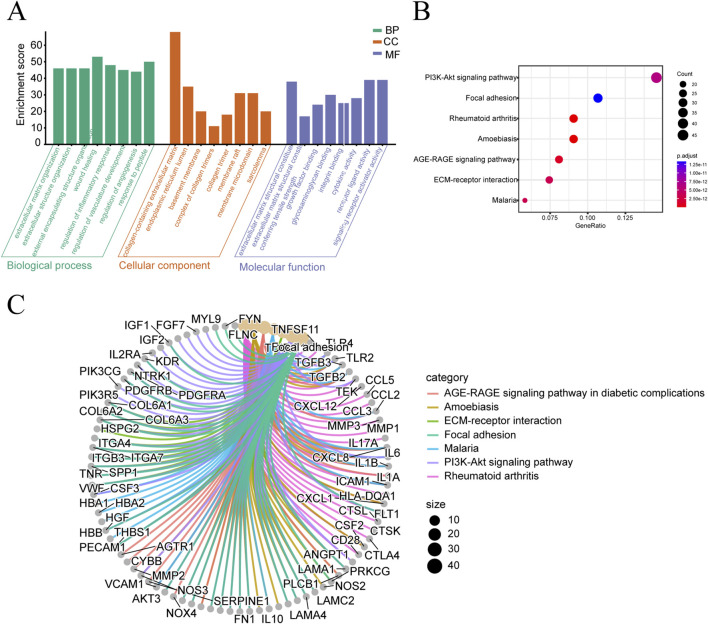
GO and KEGG enrichment analysis of intersection targets. **(A)** GO enrichment analysis of the intersection targets. **(B)** Scatter plot of KEGG pathway enrichment analysis for the intersection targets. **(C)** Network diagram of KEGG pathway enrichment analysis for the intersection targets.

GO enrichment analysis indicated that these 415 targets were significantly associated with functions such as wound healing, extracellular matrix containing collagen, and receptor ligand activity (Figure 3A). Moreover, we selected the top 7 pathways based on count and visualized them in a bubble chart (Figure 3B). The analysis revealed that the predominant enriched pathways included the PI3K-Akt signaling pathway, focal adhesion, rheumatoid arthritis, amoebiasis, AGE-RAGE signaling in diabetic complications, ECM-receptor interaction, and malaria. The enrichment of the “malaria” pathway might result from annotation overlaps in the database (e.g., shared inflammatory genes) and requires further validation for direct relevance. Additionally, the KEGG category analysis further emphasized the close association between the 415 targets and pathways such as AGE-RAGE signaling in diabetic complications, amoebiasis, ECM-receptor interaction, focal adhesion, malaria, PI3K-Akt signaling, and rheumatoid arthritis (Figure 3C).

### Core target screening and analysis

**FIGURE 4:**
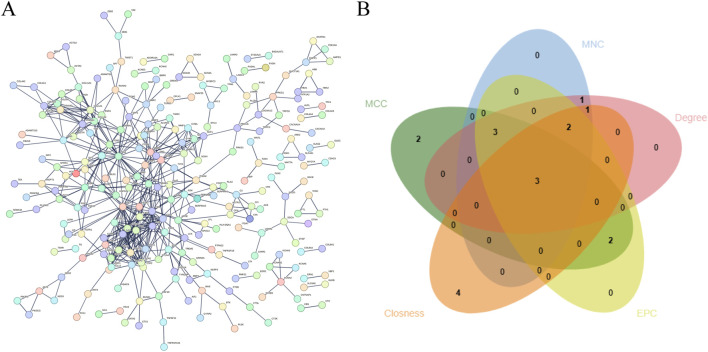
PPI of intersection targets and core target screening and analysis. **(A)** PPI network of intersection targets. Each node represents a protein, with node size proportional to its degree. Node color reflects expression levels, and edges represent protein–protein interactions, with edge thickness indicating interaction confidence. **(B)** Screening of core targets using a Venn diagram combined with the MCC, EPC, MNC, Closeness, and Degree algorithms.

To elucidate the underlying mechanism linking periodontal disease to muscle wasting and atrophy, 415 common targets were input into the STRING database to construct a protein-protein interaction (PPI) network. The resultant network and corresponding details are presented in Figure 4A. To identify key targets, a comprehensive analysis of the PPI network was performed using Cytoscape 3.9.0. Specifically, the MCC, EPC, MNC, Closeness, and Degree algorithms integrated within Cytoscape plugins were employed to screen the top 10 targets from the network. Intersection analysis of the outcomes generated by these five algorithms identified three core targets: IL-6, IL-10, and IL-1β (Figure 4B). These core targets exhibit high topological importance in the network, as evidenced by their robust scores across all five algorithms. Notably, IL-6 achieved the highest scores among the five algorithms. These findings confirm the core roles of these targets in mediating the association between periodontal disease and muscle wasting and atrophy (Table 4).

**TABLE 4:** Scores for various parameters of IL-10, IL-6, and IL-1β.

Name	MCC Score	EPC score	MNC score	Clseness score	Degree score
IL-6	731,493	41.323	26	83.75119	29
IL-10	731,187	39.596	20	71.75595	21
IL-1β	731,159	39.542	19	76.43452	22

### Evaluation of immune cell infiltration

**FIGURE 5:**
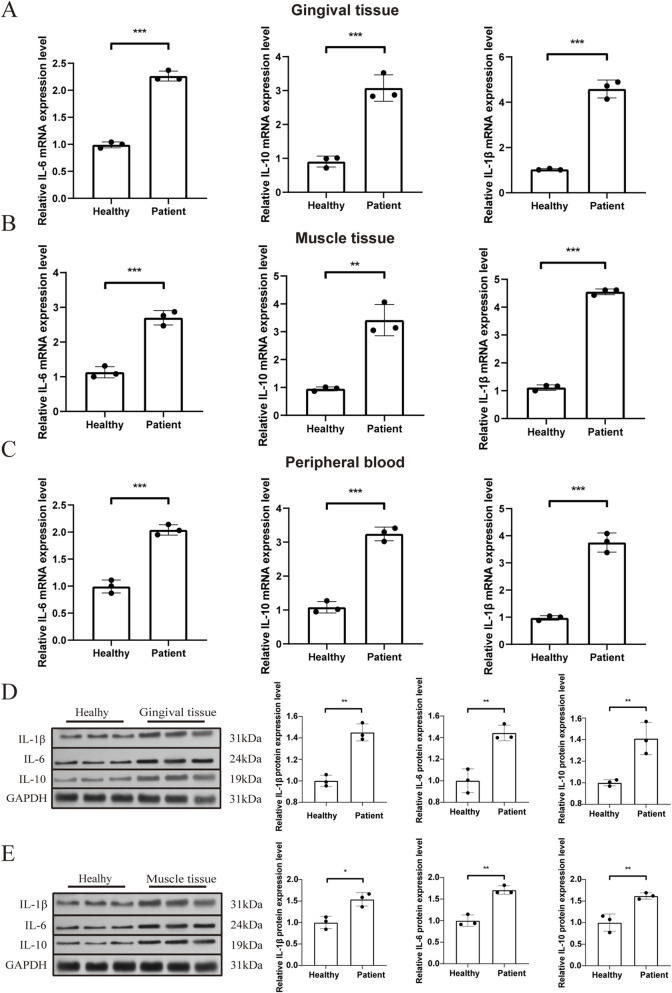
Evaluation of immune cell infiltration using the CIBERSORT algorithm. **(A)** Bar plot illustrating the relative abundance (percentage) of 22 immune cell subtypes in healthy controls versus periodontitis patients. Each color represents a specific cell type. **(B)** Box plots comparing the infiltration proportions of differentially abundant immune cells between the control and periodontitis groups. Statistical significance was evaluated using the Wilcoxon rank-sum test (*p < 0.05, **p < 0.01, ***p < 0.001). **(C)** Heatmap displaying the Spearman correlation matrix among the differentially infiltrated immune cells. Red indicates positive correlation, while blue indicates negative correlation. **(D)** Network diagram illustrating the Spearman correlations between the core cytokines (IL-10, IL-1β, IL-6) and specific immune cell subsets. Node size represents the P-value, and edge color indicates the correlation coefficient.

Given the shared dependence of periodontitis and muscle atrophy on dynamic immune microenvironment regulation, this study employed the CIBERSORT algorithm to profile the relative infiltration levels of 22 immune cell types in periodontitis and control samples. The aim was to elucidate differential immune composition and potential regulatory interactions. As shown in Figure 5A, the immune cell infiltration profiles differed markedly between groups. In controls, resting CD4 memory T cells, resting dendritic cells, and resting NK cells constituted the predominant subsets. In contrast, periodontitis samples were characterized by a higher abundance of resting CD4 memory T cells, plasma cells, and neutrophils, indicating a disease-specific remodeling of the core immune cellular landscape. Further analysis identified ten immune cell types with significantly different infiltration levels between the periodontitis and control groups, including activated mast cells, activated NK cells, CD8 T cells, M0 macrophages, naive B cells, neutrophils, plasma cells, resting dendritic cells, resting mast cells, and follicular helper T cells (Figure 5B). These findings directly demonstrate that periodontitis substantially reshapes the local immune cell infiltration pattern.

Spearman correlation analysis revealed significant synergistic or antagonistic relationships among these differentially infiltrated immune cells (Figure 5C). Activated mast cells showed a strong positive correlation with naive B cells (r = 0.70, *p* < 0.05), whereas follicular helper T cells were strongly negatively correlated with plasma cells (r = −0.73, *p* < 0.05), and activated mast cells were strongly negatively correlated with resting mast cells (r = −0.85, *p* < 0.05). This suggests that differentially infiltrating immune cells may cooperatively regulate the periodontal immune microenvironment through specific co-infiltration patterns.

Additionally, correlation analyses assessing the relationships between key cytokines (IL-10, IL-1β, IL-6) and immune cells further revealed distinct interaction profiles (Figure 5D). IL-10 showed a positive correlation with plasma cells (r = 0.65, *p* < 0.01) and a negative correlation with resting dendritic cells (r = −0.73, *p* < 0.001). IL-1β correlated positively with neutrophils (r = 0.71, *p* < 0.001) and negatively with resting dendritic cells (r = −0.77, *p* < 0.001). Similarly, IL-6 was positively correlated with plasma cells (r = 0.61, *p* < 0.01) and negatively correlated with resting dendritic cells (r = −0.80, *p* < 0.001). These results suggest that during periodontitis, immune cells may participate in modulating local inflammatory responses and maintaining immune homeostasis through the regulation of key cytokines.

### Validation of core target expression levels by qRT-PCR and western blotting

**FIGURE 6:**
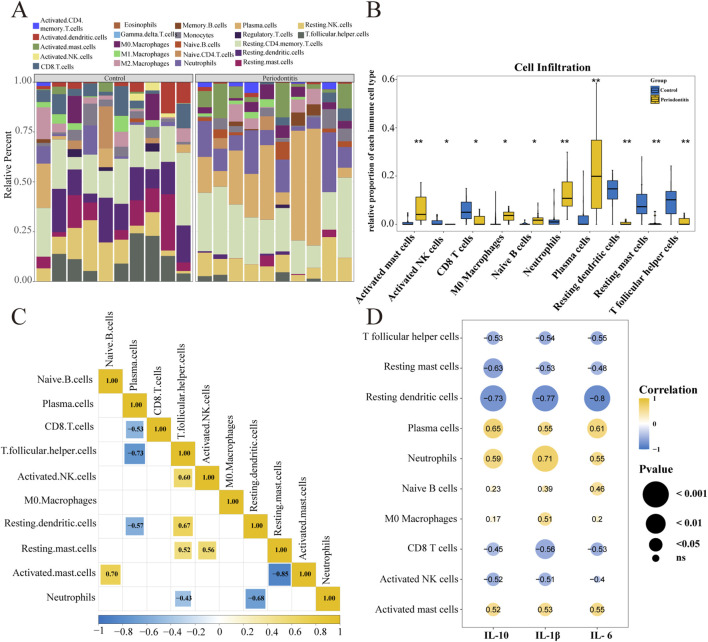
Validation of core target expression levels by qRT-PCR and Western blotting. **(A)** mRNA expression of core targets in gingival tissues from patients with periodontal disease. **(B)** mRNA expression of core targets in muscle tissues from patients with muscle wasting and atrophy. **(C)** mRNA expression of core targets in peripheral blood from patients with periodontal disease and muscle atrophy. Healthy individuals served as controls. **(D)** Protein expression of core genes in gingival tissues from patients with periodontal disease. **(E)** Protein expression of core genes in muscle tissues from patients with muscle wasting and atrophy. The sample size was n = 3 for gingival, muscle, and peripheral blood specimens in both patient and healthy control groups. GAPDH was used as an internal control. *p < 0.05, **p < 0.01.T., **p < 0.01, ***p < 0.001.

The expression levels of major inflammatory factors were quantified using qRT-PCR to verify the findings. Compared with healthy controls, mRNA levels of IL-6, IL-10, and IL-1β were notably elevated in muscle tissue (muscle wasting and atrophy patients), peripheral blood (comorbid patients), and gingival tissue (periodontal disease patients) (*p* < 0.05) (Figures 6A–C). Western blotting demonstrated a comparable pattern, with protein levels of IL-6, IL-10, and IL-1β significantly elevated (*p* < 0.05). In muscle tissue, the protein expression of the three core targets was significantly higher in muscle-wasting and atrophy patients compared to healthy controls (Figure 6D). A similar pattern was seen in the gingival tissue. In comparison to healthy controls, individuals with periodontal disease exhibited substantially higher protein expression of the core targets (Figure 6E).

### Prediction of drugs that can regulate target genes based on the coremine database

**FIGURE 7:**
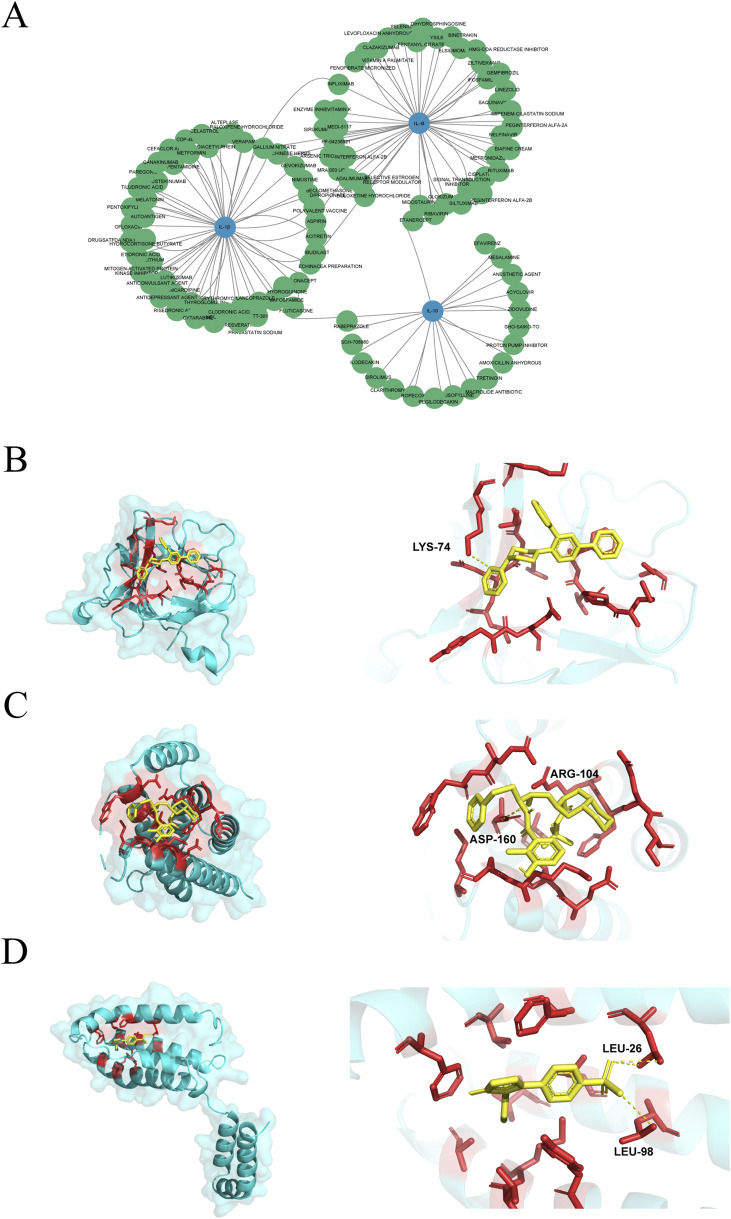
Drug prediction network and molecular docking of core targets. **(A)** Drug-target-disease interaction network, with IL-10, IL-1β, and IL-6 (central nodes) connected to candidate compounds (green) and diseases (orange). **(B–D)** The result of Molecular docking: **(B)** TT-301 and IL-1β (−7.8 kcal/mol); **(C)** Nelfinavir and IL-6 (−6.9 kcal/mol); **(D)** Rofecoxib and IL-10 (−7.8 kcal/mol).

To explore the therapeutic potential underlying the association between periodontitis and muscle atrophy, IL-10, IL-1β, and IL-6 were identified as core regulatory targets. A systematic drug prediction was performed, and a drug–target–disease interaction network was constructed using Cytoscape. The network analysis revealed that IL-10 was associated with 20 compounds and 197 diseases, IL-1β with 51 compounds and 253 diseases, and IL-6 with 49 compounds and 365 diseases, resulting in a network comprising 457 nodes and 621 edges (Figure 7A).

Subsequently, to evaluate the binding potential of candidate drugs to these targets, molecular docking was conducted. Compounds were selected based on the top five interaction scores in DGIdb for each target protein, including Acyclovir, Amoxicillin, Lisofylline, Rofecoxib, Sho-saiko-to, D-Glucosamine, Nimustine, Pentamidine, Tiludronic Acid, TT-301, Levofloxacin, Linezolid, Metronidazole, Nelfinavir, and Vitamin K, retrieved from the PubChem database (Table 5).

**TABLE 5:** Molecular docking results of candidate compounds with target proteins.

Target	PDB	Compound	PubChem	Binding energy(kcal/mol)
IL-10	1ILK	Acyclovir	135398513	−5.1
IL-10	1ILK	Amoxicillin	3613	−6.5
IL-10	1ILK	Lisofylline	501,254	−5.9
IL-10	1ILK	Rofecoxib	5,090	−7.8
IL-10	1ILK	Sho-saiko-to	5281605	−7.1
IL-1β	5R7W	D-Glucosamine	439,213	−4.7
IL-1β	5R7W	Nimustine	39,214	−5.6
IL-1β	5R7W	Pentamidine	4,735	−6.0
IL-1β	5R7W	Tiludronic Acid	60,937	−6.0
IL-1β	5R7W	Tt-301	54576073	−7.8
IL-6	1ALU	Levofloxacin	149,096	−6.7
IL-6	1ALU	Linezolid	441,401	−6.0
IL-6	1ALU	Metronidazole	4,173	−4.5
IL-6	1ALU	Nelfinavir	64,143	−6.9
IL-6	1ALU	Vitamin K	5280483	−4.8

Molecular docking was performed using the CB-Dock2 online platform, considering a binding energy < −5.0 kcal/mol as the threshold for effective binding activity. As shown in Figures 7B,D, Rofecoxib displayed the highest binding affinity for IL-10 (−7.8 kcal/mol), followed by TT-301 for IL-1β (−7.8 kcal/mol) and Nelfinavir for IL-6 (−6.9 kcal/mol).

## Discussion

Periodontal disease is a disorder characterized by the destruction of periodontal tissues due to chronic inflammation (Kinane et al., 2017), and chronic systemic inflammation is also one of the core driving mechanisms of muscle atrophy (Ji et al., 2022). Although there is a connection between the two (Bertolini and Clark, 2023), the causal relationship between them remains unclear. In recent years, Mendelian randomization studies have provided new ideas for revealing their causal directions at the genetic level (Xiao et al., 2024; Zhang et al., 2023). This study systematically evaluated the causal relationship between periodontal disease and muscle atrophy using five different MR Methods. The results suggest that periodontal disease may have a potential causal effect on muscle atrophy. It is worth noting that the inverse variance weighted (IVW) method yielded statistically significant results (*p* = 0.024, OR = 1.873), indicating that periodontal disease may be a potential risk factor for muscle atrophy. Although the IVW method showed statistical significance, other MR Methods did not reach significance, suggesting that this association still requires further verification by more independent cohorts. These observations provide additional genetic support for the previously proposed hypothesis about the association between these two situations. These observations provide additional genetic support for the previously proposed hypothesis about the association between these two situations.

Through cross-analysis of genetic datasets related to periodontal disease and muscle atrophy, this study identified 415 Intersection genes, suggesting a certain association between the two diseases. Through PPI network analysis and cross-validation in combination with five topological algorithms, IL-6, IL-10, and IL-1β were finally determined as the core targets. It is worth noting that *in vitro* experiments have also verified that the mRNA and protein expression levels of these three cytokines are significantly upregulated in the gingival tissue of patients with periodontitis, the muscle tissue of patients with muscle atrophy, and the peripheral blood of patients with comorbidities of the two diseases, suggesting that they play an irreplaceable role in the periodontitis-muscle atrophy regulatory network.

At the molecular level, IL-6, a classic pleiotropic inflammatory cytokine, exhibited the highest topological significance in this study and appears to function as a critical molecular link between periodontitis and muscle atrophy. Within periodontal tissues, pathogens such as *Porphyromonas gingivalis* activate the NF-κB signaling pathway in macrophages and fibroblasts, leading to increased IL-6 expression, which exacerbates periodontal tissue destruction and disrupts the local immune microenvironment (Uemura et al., 2022). After entering the systemic circulation, IL-6 enhances adaptive immunity by promoting B-cell differentiation into plasma cells, while simultaneously suppressing the antigen-presenting function of dendritic cells, thereby modulating innate immunity. This dual action ultimately disrupts immune homeostasis and amplifies chronic inflammatory responses. These mechanistic insights align with the immune infiltration results obtained in this study, which demonstrated a significant positive correlation between IL-6 levels and plasma cells (r = 0.61, *p* < 0.01) and a strong negative correlation with resting dendritic cells (r = −0.80, *p* < 0.001). Elevated systemic IL-6 further promotes the development of muscle atrophy. Previous studies have confirmed that IL-6 upregulates the expression of atrophy-related genes, including Atrogin-1 and MuRF-1, via activation of the JAK/STAT3 signaling pathway (Huang et al., 2020; Bonetto et al., 2012). Furthermore, KEGG enrichment analysis revealed significant enrichment of the PI3K-Akt pathway, suggesting that IL-6 may promote muscle protein catabolism and loss of muscle mass by inhibiting the key anabolic PI3K-Akt-mTOR axis and concurrently activating the ubiquitin-proteasome degradation system. Together, these processes contribute to a vicious cycle characterized by local infection, immune imbalance, systemic inflammation, and muscle wasting. This proposed mechanism is consistent with earlier reports indicating that inflammatory mediators such as IL-6 and TNF-α can inhibit downstream Akt signaling by reducing PI3K phosphorylation or enhancing phosphatase PTEN activity (Srivastava and Singla, 2023), thereby interfering with muscle protein synthesis, accelerating myofiber degradation, and ultimately driving the pathological progression of muscle atrophy.

IL-1β, a pivotal initiator of the inflammatory response, is released by activated macrophages during the early phase of periodontitis. Beyond its role in triggering local tissue injury, it functions as a core regulator of immune cell infiltration. Within the periodontal region, IL-1β enhances the resorption of the periodontal ligament and alveolar bone by upregulating the expression of matrix metalloproteinases (MMPs) (Song et al., 2019). As a potent chemotactic signal, its influence extends to systemic immune modulation. Our immune infiltration analysis revealed elevated expression of IL-1β in atrophic muscle tissues, which correlated strongly with neutrophil infiltration (r = 0.71, *p* < 0.001). These findings suggest that IL-1β likely promotes muscle atrophy (Babut et al., 2024), directly or indirectly, by mediating chemokine release and recruiting neutrophils into muscle tissue (Nakanishi et al., 2022). Moreover, IL-1β can upregulate other pro-inflammatory factors such as TNF-α and IL-6, establishing an inflammatory amplification cascade that exacerbates muscle damage and disrupts the immune microenvironment. Previous studies have further demonstrated that IL-1β synergistically inhibits the IGF-1/PI3K/Akt pathway, a key anabolic signaling axis in muscle (Strle et al., 2008). Notably, a synergistic interaction appears to exist between IL-1β-driven neutrophil infiltration and IL-6-mediated plasma cell activation, collectively driving the progression from localized periodontal lesions to systemic muscle atrophy.

IL-10 is a central regulator of immune homeostasis, chiefly through its inhibition of pro-inflammatory cytokines such as IL-6 and IL-1β (Ahmad et al., 2000). Interestingly, our investigation revealed a concomitant and significant elevation in the levels of IL-6, IL-1β, and IL-10 in the serum and periodontal tissues of periodontitis patients compared to healthy controls, pointing to a possible breakdown in immunoregulatory balance. This observation was further substantiated by immune infiltration analysis, which showed a significant positive correlation between IL-10 and plasma cells (r = 0.65, *p* < 0.01), thereby implicating IL-10 in promoting B-cell differentiation and humoral immunity. In parallel, a strong negative correlation was found between IL-10 and resting dendritic cells (r = −0.73, *p* < 0.001), aligning with its established role in suppressing innate immune activation. Although the pleiotropic immunomodulatory functions of IL-10 are critical for maintaining homeostatic equilibrium, its fundamental ability to restrain pro-inflammatory cytokine production seems to be compromised within the persistent inflammatory environment of periodontitis. Notably, while previous studies have attributed a protective role to IL-10 in preserving muscle integrity, demonstrated, for instance, by its local administration, mitigating age-related muscle regenerative decline in murine models (Dagdeviren et al., 2016). The elevated IL-10 levels observed in this study were insufficient to counterbalance the immune dysregulation and inflammatory damage mediated by IL-6 and IL-1β. This suggests that in the pathological context of periodontitis, a significantly dysregulated local inflammatory microenvironment may facilitate the systemic dissemination of inflammatory signals, ultimately perturbing the metabolic homeostasis of distal skeletal muscle.

These bioinformatics findings provide a direct mechanistic link to the Mendelian randomization results. The MR analysis demonstrated that genetically predicted periodontal disease causally increases the risk of muscle wasting and atrophy. The 415 overlapping genes identified through bioinformatics, with IL-6, IL-1β, and IL-10 as core hub nodes in the PPI network, are enriched in the PI3K-Akt signaling pathway. This convergence indicates that the genetic predisposition to periodontal disease likely drives chronic systemic inflammation, which in turn activates PI3K-Akt-mediated protein catabolism in skeletal muscle—thereby offering a plausible biological mechanism underlying the observed causal association.

Furthermore, the drug prediction analysis based on the core targets provides new therapeutic insights for the cross-disease management of periodontitis and muscle atrophy. For example, rofecoxib, a selective cyclooxygenase-2 (COX-2) inhibitor with proven anti-inflammatory effects in periodontitis (Gopikrishna and Parameswaran, 2003), may enhance its efficacy by targeting IL-10, as suggested by molecular docking. Meanwhile, TT-301, a novel immunomodulator previously used mainly in autoimmune diseases (James Michael et al., 2012), shows high affinity for IL-1β, offering a promising direction for anti-inflammatory treatment of muscle atrophy. In addition, nelfinavir, an antiviral protease inhibitor, has recently been found to possess anti-inflammatory and muscle-protective properties (Meena et al., 2025). This study further elucidates its mechanism by associating it with the regulation of IL-6. Notably, conventional periodontal treatments such as amoxicillin and metronidazole were also identified in the prediction. The connection between these drugs and the core targets suggests that traditional periodontal therapy may indirectly protect against muscle atrophy by modulating the IL-6 and IL-1β pathway.

It should be noted that the present study has several limitations. First, the GWAS summary datasets used in this study were not stratified by age, diabetes, cardiovascular diseases, or inflammation status; therefore, residual confounding cannot be completely excluded. Future stratified MR analyses are warranted to validate the stability of the causal. Second, both GWAS datasets were derived exclusively from European-ancestry populations, limiting generalizability to other ethnic groups (e.g., Asian populations) and to different disease stages or severities. Third, we experimentally validated key cytokines (IL-6, IL-1β, and IL-10) in a small clinical cohort with 3 samples per group. Although qRT-PCR and Western blot results were consistent with bioinformatic predictions, the small sample size reduced statistical power and reliability. Thus, further validation in a larger clinical cohort is required. Fourth, drug prediction and molecular docking in this study were only based on *in silico* exploratory screening without cellular, animal, or clinical experimental validation; therefore, the relevant findings have no direct clinical application value. Finally, although we performed MR-Egger regression and Cochran’s Q test to assess pleiotropy and heterogeneity, we did not conduct MR-PRESSO analysis. MR-PRESSO is particularly valuable for detecting and correcting individual outlier SNPs that may drive horizontal pleiotropy. While our MR-Egger intercept test suggested no significant directional pleiotropy, the lack of MR-PRESSO is a limitation, and future studies with larger genetic datasets are warranted to apply this method for a more comprehensive validation of our findings.

## Conclusion

This study initially explored the potential causal relationship between periodontal disease and muscle atrophy, suggesting that chronic systemic inflammation is the central mediator. Specifically, IL-6, IL-1β, and IL-10 were identified as key regulatory molecules within this pathological network. Pathway enrichment analysis further suggested that the PI3K–Akt signaling axis acts as a critical functional hub in this cross-disease interaction. Notably, immune infiltration analysis revealed significant correlations between these cytokines and specific immune cell subsets, indicating that dysregulation of the local immune microenvironment in periodontitis may propagate systemic inflammation and accelerate muscle atrophy. *In vitro* assays confirmed the coordinated dysregulation of these inflammatory mediators under pathological conditions. Furthermore, systematic drug prediction and molecular docking identified several candidate compounds with translational potential for cross-disease intervention, including rofecoxib, TT-301, and nelfinavir. These findings illuminate the pathophysiological mechanism by which periodontal disease may systemically exacerbate muscle atrophy, highlighting the imperative for integrated treatment strategies that encompass oral health to preserve musculoskeletal function and improve overall patient wellbeing.

## Data Availability

The original contributions presented in the study are included in the article/supplementary material, further inquiries can be directed to the corresponding author.
